# Gold Nanoparticles as a Photothermal Agent in Cancer Therapy: The Thermal Ablation Characteristic Length

**DOI:** 10.3390/molecules23061316

**Published:** 2018-05-31

**Authors:** Thomas Grosges, Dominique Barchiesi

**Affiliations:** Group for Automatic Mesh Generation and Advanced Methods (Gamma3 UTT-INRIA), University of Technology of Troyes, 12 rue Marie Curie, CS 42060, F-10004 Troyes CEDEX, France; dominique.barchiesi@utt.fr

**Keywords:** numerical approximation and analysis, metallic nanostructures, medical optics and biotechnology, simulations, thermal agent, photothermal effects

## Abstract

In cancer therapy, the thermal ablation of diseased cells by embedded nanoparticles is one of the known therapies. It is based on the absorption of the energy of the illuminating laser by nanoparticles. The resulting heating of nanoparticles kills the cell where these photothermal agents are embedded. One of the main constraints of this therapy is preserving the surrounding healthy cells. Therefore, two parameters are of interest. The first one is the thermal ablation characteristic length, which corresponds to an action distance around the nanoparticles for which the temperature exceeds the ablation threshold. This critical geometric parameter is related to the expected conservation of the body temperature in the surroundings of the diseased cell. The second parameter is the temperature that should be reached to achieve active thermal agents. The temperature depends on the power of the illuminating laser, on the size of nanoparticles and on their physical properties. The purpose of this paper is to propose behavior laws under the constraints of both the body temperature at the boundary of the cell to preserve surrounding cells and an acceptable range of temperature in the target cell. The behavior laws are deduced from the finite element method, which is able to model aggregates of nanoparticles. We deduce sensitivities to the laser power and to the particle size. We show that the tuning of the temperature elevation and of the distance of action of a single nanoparticle is not significantly affected by variations of the particle size and of the laser power. Aggregates of nanoparticles are much more efficient, but represent a potential risk to the surrounding cells. Fortunately, by tuning the laser power, the thermal ablation characteristic length can be controlled.

## 1. Introduction

In the last few decades, photothermal measurement techniques and applications to photothermal therapy (PTT) have been experiencing significant developments [1,2]. Specifically, using near-infrared laser nanoabsorbers to generate a local heat source, inducing thermal ablation of cancer cells, has opened a new therapeutic area that provides more specificity and preserves surrounding tissues [3,4,5]. Such therapies are non-invasive, as they do not require surgery. Thermal ablation involves the delivery of nanoparticles by injection and illumination by using a laser to produce local heating, which destroys the tumoral cell [6,7,8,9,10]. Nanoparticles with various shapes and sizes are therefore photothermal agents (PA). A continuous waveform laser illuminates the PA that absorb a part of the electromagnetic energy, which is converted into heat. The resulting temperature elevation depends on the geometrical and material characteristics of the PA. It is expected to overpass the thermal ablation threshold while avoiding exceeding the human body temperature outside the treated cell. Under such constraints, the control of the temperature is critical.

Here, we propose to calculate the spatial distribution of temperature in and around a settlement of nanoparticles in a wide of range of parameters (diameters and laser power) and to select the solutions that address the two constraints mentioned above. Then, behavior laws can be deduced from the selected results. To calculate the temperature elevation induced by a single nanoparticle or aggregates in cells, illuminated by a laser, we use the finite element method. We solve both the electromagnetic wave and the heat partial differential equations by using a 3D adaptive remeshing scheme with an error estimator to control the accuracy of solutions in the multiscale domain of computation [11,12,13]. The numerical model includes the electromagnetic and thermal coupling of nanoparticles in the cell [13]. We deduce behavior laws that relate the maximum temperature, the particle size and the thermal ablation characteristic length (TACL). TACL is the spatial extension of temperature around the nanoparticle and can be defined as the distance to the particle over which the temperature exceeds the ablation threshold. From behavior laws, the uncertainty of the TACL is calculated as a function of both the uncertainty of the nanoparticle size and that of the laser power.

The organization of the paper is as follows. In Section 2, we detail the equations of the problem, the physical parameters and the numerical method. In Section 3, we give the results of simulations, and we discuss the ability to control the temperature and the TACL in the cell before concluding.

## 2. Photothermal Model

Absorbing media are sources of temperature elevation when illuminated by electromagnetic waves. In the present case, gold nanoparticles are absorbing electromagnetic energy and are heat sources, the density of which depends on the local electric field as follows:

(1) $$\begin{array}{c} Q\left(x\right)=\frac{\omega }{2}\epsilon_{0}Im\left(\epsilon_{r}\left(x\right)\right){\left|E(x)\right|}^{2},\;\;\;\forall x\in \Omega , \end{array}$$

where ω is the angular frequency of the incoming laser wave, $\epsilon_{0}$ is the permittivity of a vacuum and $\epsilon_{r}$ is the relative permittivity of materials. Such relative permittivity $\epsilon_{r}$ is a complex number for absorbing materials; $Im(\epsilon_{r}\left(x\right))$ is its imaginary part and is a function of the 3D spatial coordinates x in the computation domain Ω. E(x) denotes the total electric field, which is the solution of the electromagnetic partial differential equation:

(2) $$\begin{array}{c} \nabla \times \left(\frac{1}{\mu_{r}\left(x\right)}\nabla \times E\left(x\right)\right)-\frac{\omega^{2}}{c^{2}}\epsilon_{r}\left(x\right)E\left(x\right)=0,\;\;\;\forall x\in \Omega , \end{array}$$

where $\mu_{r}$ is the relative permeabilities of the materials (here $\mu_{r}=1$), *c* is the velocity of light and (∇×) is the curl operator. The resolution of Equation (2) is achieved with a radiation boundary condition [13,14,15] at the external border Γ of the computational domain Ω. This boundary condition describes the free propagation of the total electric field. The illumination laser field has amplitude:

(3) $$\begin{array}{c} E_{0}=\sqrt{\frac{8c\mu_{0}P_{w}}{\pi D_{beam}^{2}}}=\sqrt{2c\mu_{0}P_{s}}, \end{array}$$

where $\mu_{0}$ is the permeability of a vacuum, $P_{w}$ is the laser power, $D_{beam}$ is the diameter of the laser beam and $P_{s}=4P_{w}/\left(\pi D_{beam}^{2}\right)$ is the surface laser power density. The heat source produces a temperature elevation, which is governed by the heat equation:

(4) $$\begin{array}{c} \frac{\partial T(x,t)}{\partial t}-\frac{1}{\rho \left(x\right)C_{p}\left(x\right)}\nabla \cdot \left(\kappa (x)\nabla T(x,t)\right)=\frac{Q(x)}{\rho \left(x\right)C_{p}\left(x\right)},\;\;\;\;\;\forall x\in \Omega , \end{array}$$

where *t* is the time, *T* is the temperature, ρ is the volumic mass density of material, $C_{p}$ is the specific heat capacity, κ is the thermal conductivity and *∇* and (∇·) are the gradient and the divergence operators. The material parameters and the temperature are functions of the 3D spatial coordinates x.

Therefore, the resolution of the full problem requires solving the heat equation (Equation (4)) with a source *Q* produced by the incoming electromagnetic illumination (Equation (1)) with the boundary condition $T\left(x,t\right)=T_{0}$ at the boundary of the computational domain (i.e., ∀x∈Γ). $T_{0}$ will be the body temperature in further calculations. The resolution of the weak coupled photothermal problem gives the spatio-temporal distribution of the temperature in the computational domain. For an illumination duration *t* longer than the characteristic thermic time of materials $\tau_{therm}$ (i.e., $\tau_{therm}\approx 1$ ms), the resolution of the problem (Equation (4)) converges to the stationary solution (i.e., $t\geq \tau_{therm}$, ∂T(x,t)/∂t⟶0) and corresponds to a continuous illumination. Let us note that the characteristic time $\tau_{elec}$ for the electromagnetic problem is much shorter than thermic time ($\tau_{elec}\approx 3$ fs for a near-infrared laser illumination).

**Figure 1:**
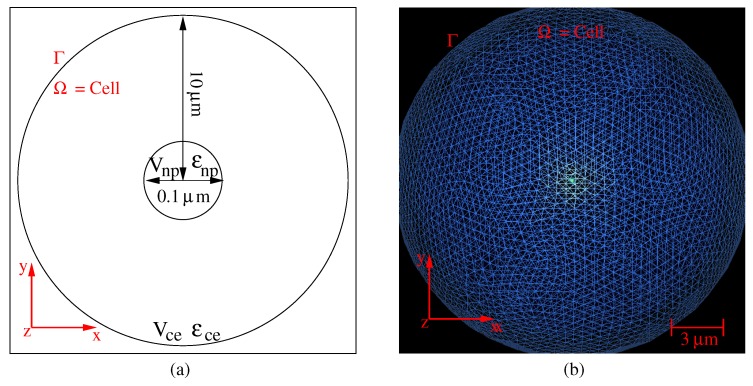
Slice of the geometry (**a**) and of the associated mesh (**b**) of a spherical nanoparticle embedded in a spherical cell.

In this context, we use a 3D numerical model for the accurate computation of the photothermal process around the gold nanoparticle embedded in a cell. Equations (2)–(4) are solved numerically through a 3D finite element method (FEM) with an adaptive remeshing scheme using an a priori error estimator based on the numerical Hessian of the solution. Such an adaptive remeshing scheme with error estimator permits one to control the accuracy of computation in the multiscale domain: the typical size of nanoparticles is a few tens of nanometers, and that of the cell is a few tens of microns. With FEM, the problem is solved in a discrete domain represented by a mesh [14,16,17], and the unknown physics quantities (here E and *T*) are computed at the nodes of the mesh by using a variational method. We use an improved 3D method, including a process of iterative remeshing [11,12,13,15], to control the error on the numerical solution. Indeed, the accuracy of the computed solutions depends on the quality of the mesh [18,19]. Therefore, the accuracy of the solutions is improved by automatically adapting the size of the mesh elements to the solution, at each step of the remeshing process [12,15,20] through adaptive loops. For the phenomenon inducing a strong variation in the calculated quantities of interest, the convergence of the numerical solution toward a stable solution only requires such mesh adaptions [11,12]. At each step of the adaption process, the approximations of the temperature *T* (Equation (4)) are calculated on the basis of the interpolation polynomials between nodes. The maximum deviation between the solution associated with the mesh and the computed solution at nodes is limited by the interpolation error (which is based on an estimation of the discrete Hessian of the solution) [21,22]. Therefore, the numerical scheme not only takes into account the shape and size of the nanoparticle, but also the local variations of both the electromagnetic field and the temperature. Such a process is obtained from the OPTIFORM software (adaptive remeshing generating isotropic or anisotropic meshes) [11,12] that governs the remeshing of the domain (with minimum and maximum element sizes set to $h_{min}=0.1$ nm and $h_{max}=500$ nm and with a tolerance on the relative error on the computed unknown physics quantities δ=0.1%). Therefore, the domain is entirely remeshed at each adaption step, and a new mesh is produced. Figure 1a,b shows a schematic of the reference problem (a single nanoparticle at the center of a spherical cell) and an example of the mesh of the domain of computation Ω.

From this numerical model, the temperature variations that are induced by the electromagnetic and thermal coupling of nanoparticles in the cell are studied. That permits one to deduce the behavior laws that relate the maximum temperature and the TACL of the spatial extension of temperature around the nanoparticle. The uncertainty of the TACL as a function of the uncertainties of the nanoparticle size and of the maximum temperature will also be given. This uncertainty could be used to deduce the sensitivity of the TACL to the statistical dispersion of particle sizes inherent in their process of fabrication.

## 3. Numerical Results and Discussion

The cell’s membrane has spatial extension from −10μm–10μm, and the gold nanoparticles have diameters varying in [50; 150] nm. Therefore, the problem is a multi-scale problem as the ratio of the volume of the cell to that of nanoparticles $V_{ce}/V_{np}\in \left[2.4;64.2\right]\times 10^{6}$. The cell’s volume is illuminated by an electromagnetic wave at λ=830 nm. This wavelength is chosen to be in the near-infrared region where the laser irradiation can both penetrate soft tissues for many centimeters deep with low absorption and overlap the absorption band of gold nanoparticles [8,23]. The illumination laser beam has power $P_{w}$ in [0.5; 2.0] W and a beam diameter $D_{beam}=0.15$ mm. The body temperature on the boundary of the cell is $T_{0}=37.0{\;}^{\circ }$C (i.e., 310.15 K). The parameters of Equations (1)–(4) for the electromagnetic and thermic problems are given in Table 1 [24]. All materials are considered isotropic and homogeneous.

In the following, we first consider a single nanoparticle, located at the center of the cell, before investigating an aggregate of nanoparticles in Section 3.4. To characterize the temperature elevation that is induced by illumination, we study both the influence of the laser power and of the volume of the nanoparticle. The volume of nanoparticle is a relevant parameter, the thermal source being a function of the coordinates in the nanoparticle (Equation (1)).

The constraints for thermal ablation are: First, the temperature increase must exceed the thermal ablation threshold $T_{ce,abl}$ in the cell. Second, a sharp decrease of the temperature around the nanoparticle is expected in order to protect the neighboring cells $T_{0}$. The higher the maximum temperature in the nanoparticle is, the larger the TACL will be. Therefore, to ensure the constraint of the conservation of the body temperature in the surroundings of the diseased cell, a balance between the laser power and the particle size inducing a maximum temperature in the nanoparticle should be found. To satisfy these constraints, we must consider that the maximum of the temperature in the particle $T_{np,max}$ must belong to the following interval $T_{np,max}\in \left[42;52\right]{\;}^{\circ }C$. Therefore, the TACL can be defined as:

(5) $$\begin{array}{c} d_{ce,abl}=d\left(x,T_{np,max},T_{ce,abl},T_{0}\right). \end{array}$$

**Figure 2:**
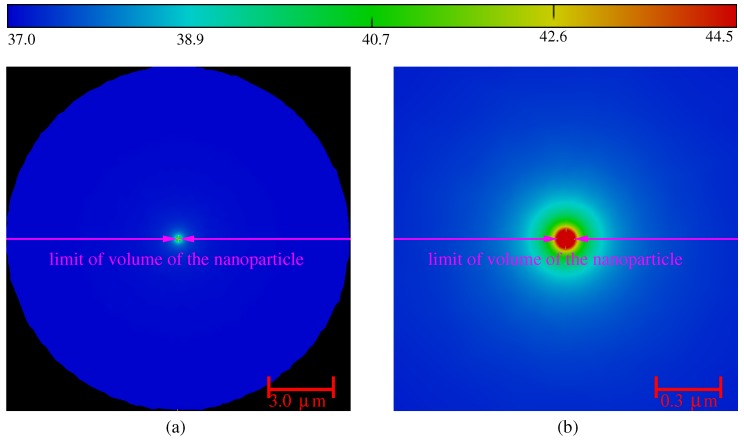
Temperature in the z=0 plane, in the whole computational domain (**a**) and zoom around the gold nanoparticle (**b**). The volumes of the nanoparticle and the cell are: $0.52\times 10^{-3}\;\mu$m${}^{3}$ and $4.2\times 10^{3}\;\mu$m${}^{3}$, respectively. The cell’s membrane has spatial extension from −10 μm to 10 μm, and the diameter of the nanoparticle is 100 nm.

This TACL $d_{ce,abl}$ is the distance to the particle over which the temperature exceeds $T_{ce,abl}=42{\;}^{\circ }$C. The results of the computation of the temperature are illustrated in Figure 2a,b for a nanoparticle diameter of 100 nm ($V_{np}=0.52\times 10^{-3}\;\mu$m${}^{3}$) under an illumination power $P_{w}=1$ W.

The spatial variations of temperature are shown with z=0, in the whole computational domain (a) and zoomed around the nanoparticle (b). The results have been compared to the analytical solution (Mie theory for the electromagnetic problem) that exists for this reference problem with high symmetry [11,12,15]. The comparisons are in good agreement with the analytical solution, and the errors are smaller than 0.1%. Nevertheless, the degree of symmetry of the thermal source *Q* prevents deducing a simple analytical solution for the thermal problem, unless considering an arbitrary homogenization of the source in the nanoparticle. The fact remains that the point symmetry property of the temperature is checked in the FEM results. This property is congruent with the small ratio of the nanoparticle size to the TACL. The relation between the volume $V_{np}$ of the nanoparticle, the laser power $P_{w}$ and the temperature can be deduced. This first calculation shows that the typical TACL is about 25 nm around the nanoparticle.

### 3.1. Influence of the Laser Power and of the Volume of Nanoparticles

**Figure 3:**
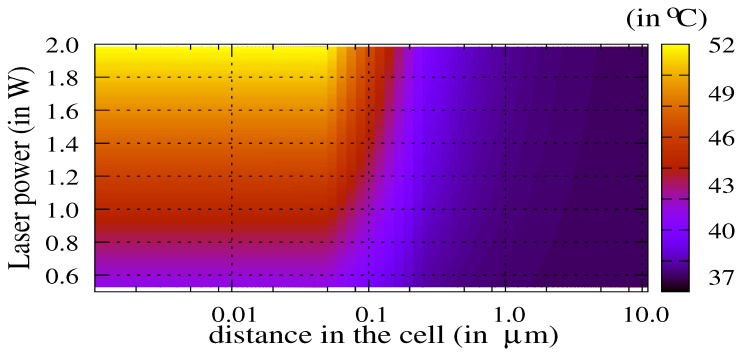
Map of the temperature (in ${}^{\circ }$C) as a function of both the distance to the center of nanoparticle in the cell (i.e., distance ≤10μm) and the incident laser power ($P_{w}\in \left[0.5;2.0\right]$ W). The diameter of the gold nanoparticle is $D_{np}=100$ nm ($V_{np}=0.52\times 10^{-3}\;\mu$m${}^{3}$).

Figure 3 shows the map of the temperature in the cell as a function of the laser power $P_{w}\in \left[0.5;2.0\right]$ W and as a function of the distance from the center of the nanoparticle.

**Figure 4:**
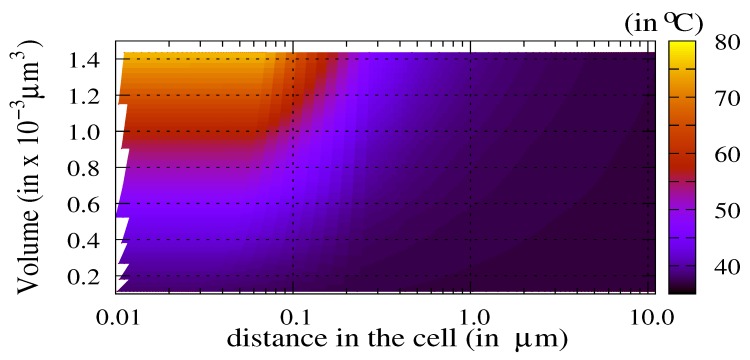
Temperature (in ${}^{\circ }$C) as a function of the distance from the center of the nanoparticle in the cell (i.e., distance ≤10μm) and as a function of the volume of nanoparticle ($V_{np}\in \left[0.1;1.5\right]\times 10^{-3}\;\mu$m${}^{3}$). The diameter of the nanoparticle varies from 50 nm–150 nm, and the laser power is $P_{w}=1.0$ W.

The TACL weakly depends on the laser power above a threshold of 0.8 W for this particle diameter. This threshold is the efficiency limit of the nanoparticles for thermal ablation. Below this threshold, the particle is inactive. Figure 4 shows the temperature in the cell as a function of both the volume of the nanoparticle $V_{np}\in \left[0.1;1.5\right]\times 10^{-3}\;\mu$m${}^{3}$ and the distance from the center of the nanoparticle.

The laser power is $P_{w}=1.0$ W. The weak dependence on the distance is observed proviso with respect to the threshold for active particles: the volume of nanoparticles must exceed a fair value of about $0.4\times 10^{-3}\;\mu$m${}^{3}$ for this laser power. We must underline that the permittivity value is assumed as constant as a function of the particle’s size (bulk value). Therefore, for small nanoparticles, the temperature values could be slightly different due to a variation of the permittivity $\epsilon_{r}$. For larger nanoparticles, the bulk value is a good approximation. By controlling the target temperature $T_{np,max}$ in the nanoparticle through the choice of both the volume $V_{np}$ and the laser power $P_{w}$, it becomes possible to evaluate the TACL (Equation (5)).

### 3.2. The Thermal Ablation Characteristic Length

**Figure 5:**
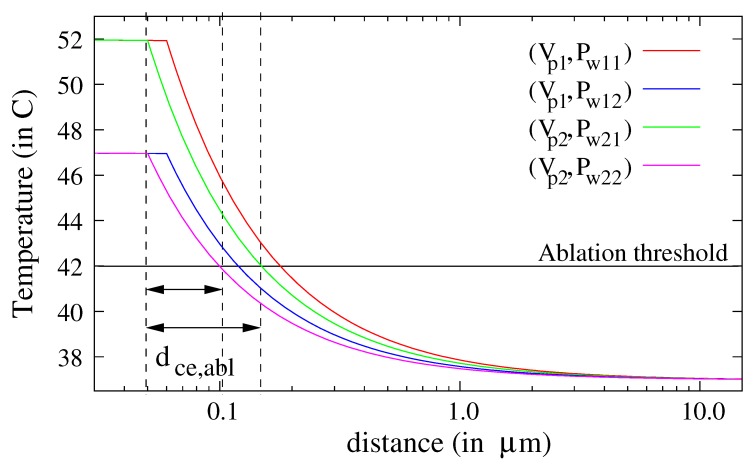
Evolution of the temperature in the cell for two volumes of nanoparticles: $V_{p1}=0.91\times 10^{-3}\;\mu$m${}^{3}$, $V_{p2}=0.52\times 10^{-3}\;\mu$m${}^{3}$; and four laser powers: $P_{w11}=0.9$ W, $P_{w12}=0.6$ W, $P_{w21}=1.9$ W, $P_{w22}=1.3$ W. The ablation threshold is shown.

To evaluate the TACL (Equation (5)), we first consider intervals of variation of the laser power and the volume of nanoparticles, producing temperature elevations of 47 and 52 ${}^{\circ }$C. The decrease of the temperature in the cell is plotted in Figure 5 for two volumes of nanoparticle and four laser powers ($V_{p1}=0.91\times 10^{-3}\;\mu$m${}^{3}$, $D_{np1}=120$ nm, $V_{p2}=0.52\times 10^{-3}\;\mu$m${}^{3}$, $D_{np2}=100$ nm, $P_{w11}=0.9$ W, $P_{w12}=0.6$ W, $P_{w21}=1.9$ W, $P_{w22}=1.3$ W). The temperature tends rapidly to the temperature of the body $T_{0}=37{\;}^{\circ }C$ in the cell.

**Figure 6:**
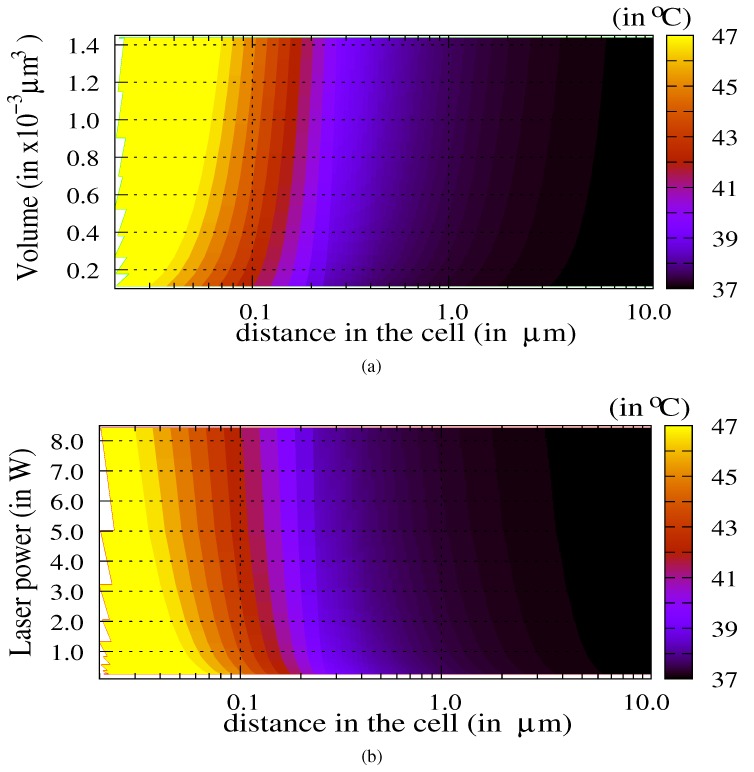
Maps of the temperature in the cell as a function of the nanoparticle volume (**a**) and of the laser power (**b**). The laser power and the volume of the nanoparticle are adapted respectively in order to produce a selected maximum temperature in the particle $T_{np,max}=47{\;}^{\circ }C$.

The intersections between the ablation threshold (horizontal line at $T=42{\;}^{\circ }C$) and the curves of the temperature reveal that the TACL varies between 50 nm and 120 nm, even if the laser power is increased by 50% and the volume of the nanoparticle by 100%. Therefore, a classical calculation of the propagation of uncertainties may be adequate to evaluate the sensitivity of the TACL to the laser power and the nanoparticles’ diameters dispersion [25]. To check the validity of our claim, we also evaluate the variations of the TACL (Equation (5)) at a central temperature of 47 ${}^{\circ }$C: $d_{ce,abl}=d\left(x,T_{np,max}=47\;{}^{\circ }C,T_{ce,abl},T_{0}\right)$. For this, we first select the results of computations that fulfill the condition: $T_{np,max}=47\;{}^{\circ }C$. Therefore, the pairs of parameters (volume of nanoparticles $V_{np}$ and laser power $P_{w}$) that were used can be deduced. Figure 6a,b shows the variations of temperature in the cell for all these solutions.

Let us note that the trends of Figure 6a,b are inverse in $V_{np}$ and $P_{w}$: an increasing volume induces a decreasing laser power for fixed value $T_{np,max}$. These 2D maps of temperature confirm the weak dependence of the TACL as a function of the volume of the nanoparticle and of the laser power. Therefore, the sensitivity of this TACL with the dispersion of these parameters cannot be deduced directly. To evaluate this sensitivity, we propose an analysis of uncertainties.

### 3.3. Sensitivity Analysis

To deduce the sensitivity of the TACL with the laser power and with the size of nanoparticles, we first calculate the sensitivity of the maximum temperature. An approximation of such maximum temperature $T_{np,max}$ in the nanoparticle can be deduced from Equation (4) and from the boundary conditions at the interface between the nanoparticle and the cell:

(6) $$\begin{array}{c} T_{np,max}\approx T_{0}+\frac{Q}{\rho_{np}C_{p,np}}\frac{\rho_{ce}C_{p,ce}}{\kappa_{ce}}V_{np}\left[\frac{D_{np}}{2S_{np}}-\frac{D_{ce}}{2S_{ce}}\right]\leq T_{0}+\frac{Q}{\rho_{np}C_{p,np}}\frac{\rho_{ce}C_{p,ce}}{\kappa_{ce}}\frac{V_{np}D_{np}}{2S_{np}}, \end{array}$$

where *V* is the volume, *S* the surface of the boundary, *D* the diameter and the subscripts np and ce refer to the nanoparticle and to the cell, respectively. *Q* is the mean of the heat source (Equation (1)) in the volume $V_{np}$; $T_{0}$ is the temperature of the body; and material parameters are given in Table 1. By inverting Equation (6) for the nanoparticle size $D_{np}$ (through $V_{np}$, $S_{np}$ and $D_{np}$), the laser power density $P_{s}$ (or $P_{w}$ through *Q* in Equations (1) and (3)) can be tuned for fixed values of $T_{np,max}$:

(7) $$\begin{array}{ccc} \left(T_{max,np}-T_{0}\right) & = & \alpha \beta P_{s}\bar{{\left|E/E_{0}\right|}^{2}}D_{np}^{2}/12, \end{array}$$

(8) $$\begin{array}{ccc} P_{s} & = & \frac{12(T_{max,np}-T_{0})}{\alpha \beta D_{np}^{2}\bar{{\left|E/E_{0}\right|}^{2}}}, \end{array}$$

with $\alpha =\left(\rho_{ce}C_{p,ce}\right)/\left(\rho_{np}C_{p,np}\right)$, $\beta =\omega Im(\epsilon_{r})/c$ and $\bar{{\left|E/E_{0}\right|}^{2}}$ is the mean normalized electric intensity in the nanoparticle’s volume. Assuming a legitimate assumption of the dispersion of nanoparticle diameters and of laser power (due to the penetration in tissues), the uncertainty $u(T_{np,max})$ on the target maximum temperature in the nanoparticle helps to evaluate the sensitivity of the temperature elevation to the variations of the diameter and the illumination power. With such an approximation and expressing $V_{np}$ and $S_{np}$ as a function of $D_{np}$ and *Q* as a function of $P_{w}$, the uncertainty over the maximum temperature $u(T_{np,max})$ can be deduced [25]:

(9) $$\begin{array}{c} u\left(T_{np,max}\right)\leq \left(T_{np,max}-T_{0}\right){\left[u_{r}^{2}\left(P_{w}\right)+4u_{r}^{2}\left(D_{np}\right)\right]}^{1/2}, \end{array}$$

where $u_{r}\left(.\right)$ are the relative uncertainties; for the values of laser power $P_{w}\approx 1$ W with uncertainty $u(P_{w})\approx 20$ mW, a nanoparticle diameter $D_{np}\approx 100$ nm with dispersion $u(D_{np})\approx 3.0$ nm and a target temperature value in the nanoparticle $T_{np,max}\approx 47$ ${}^{\circ }$C, $u(T_{np,max})\approx 0.63$ ${}^{\circ }$C. This value is small with reference to the target values $T_{np,max}\in \left[42;52\right]{\;}^{\circ }C$. This first numerical result shows that a wide range of diameter and laser power can be used to reach the target values. From Figure 5 and Figure 6, we can also deduce that the temperature in the cell is proportional to the inverse of the distance to the nanoparticle boundary. Therefore, the relation between the spatial extension of the temperature in the cell and the size of the nanoparticle is deduced:

(10) $$\begin{array}{ccc} T_{ce}\left(x\right) & = & \frac{\left(T_{np,max}-T_{0}\right)}{x}\frac{D_{np}}{2}+T_{0},\;\;\;\mathrm{for}\;x\geq D_{np}/2, \end{array}$$

(11) $$\begin{array}{ccc} d_{ce,abl} & = & \frac{\left(T_{np,max}-T_{0}\right)}{\left(T_{ce,abl}-T_{0}\right)}\frac{D_{np}}{2}-D_{np}/2=\frac{\left(T_{np,max}-T_{ce,abl}\right)}{\left(T_{ce,abl}-T_{0}\right)}\frac{D_{np}}{2}, \end{array}$$

where $d_{ce,abl}$ is the TACL (for temperatures above the ablation threshold $T_{ce,abl}=42$ ${}^{\circ }$C). Equation (11) gives the same values as observed in Figure 5: $d_{ce,abl}\in \left[50;100\right]$ nm for $D_{np}=100$ nm and $d_{ce,abl}\in \left[60;120\right]$ nm for $D_{np}=120$ nm. The uncertainty $u(d_{ce,abl})$ of the TACL in the cell is a function of the nanoparticle diameter and of the maximum temperature in the nanoparticle:

(12) $$\begin{array}{ccc} u(d_{ce,abl}) & = & d_{ce,abl}\left(\frac{T_{np,max}-T_{0}}{T_{np,max}-T_{ce,abl}}\right){\left[u_{r}^{2}\left(P_{w}\right)+{\left(\left(\frac{T_{np,max}-T_{ce,abl}}{T_{np,max}-T_{0}}\right)+2\right)}^{2}u_{r}^{2}\left(D_{np}\right)\right]}^{1/2}. \end{array}$$

For example, by considering a mean diameter $D_{np}=100\;\mathrm{nm}$ with $u_{r}\left(D_{np}\right)\approx 3.0\%$ and a power laser $P_{w}=1\;W$ with $u_{r}\left(P_{w}\right)\approx 2.0\%$, the TACL in the cell is $d_{ce,abl}\pm u\left(d_{ce,abl}\right)=\left(50.0\;\pm \;7.8\right)$ nm. The dominant contribution to the uncertainty $u(d_{ce,abl})$ comes from the $u_{r}\left(D_{np}\right)$. From these results, we can deduce that the efficiency volume of nanoparticles is $V_{ce,T>abl}\approx 5.1\times 10^{-3}\;\mu$m${}^{3}$ (i.e., $1.2\times 10^{-6}$ relative to the cell’s volume). In this efficiency volume, the temperature is larger than the ablation temperature.

So far, we have focused on the study of a single nanoparticle; nevertheless, metal nanoparticles can also aggregate by 1–15 nanoparticles/vesicles [26,27]. In the next subsection, we discuss the influence of the distance between three nanoparticles.

### 3.4. Aggregates of Nanoparticles

For aggregates, the interdistances between the nanoparticles are additional parameters of the problem. Moreover, the electromagnetic coupling between nanoparticles induces electromagnetic field enhancement and could influence the temperature elevation in the cell. However, the electromagnetic coupling occurs at a short distance between the nanoparticles ($d_{elec}\approx 50--70$ nm). On the other hand, the thermic coupling could appear if the inter-distance is typically lower than $d_{therm}\approx 1\;\mu$m (see Figure 6). Consequently, we expect to distinguish three zones of coupling:

- $d\leq d_{elec}$: electromagnetic and thermal coupling,
- $d_{elec}\leq d\leq d_{therm}$: thermal coupling,
- $d\geq d_{therm}$: no coupling.

**Figure 7:**
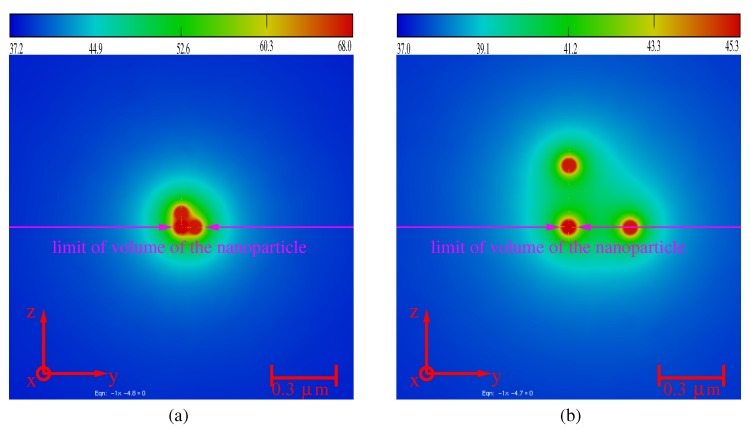
Map of the temperature in the cell for a system of three embedded nanoparticles of diameter $D_{np,i}=100$ nm. (**a**) $d_{1,2}=d_{1,3}=10$ nm: strong coupling. (**b**) $d_{1,2}=d_{1,3}=400$ nm: weak coupling.

With FEM, we can solve numerically the photothermal problem of *N* particles embedded everywhere in the cell. We compute and analyze the modifications of the temperature map induced by a system of N=3 identical nanoparticles ($D_{np}=100$ nm). Figure 7a,b shows the temperature maps in the cell for two configurations ($D_{np}=100$ nm).

**Figure 8:**
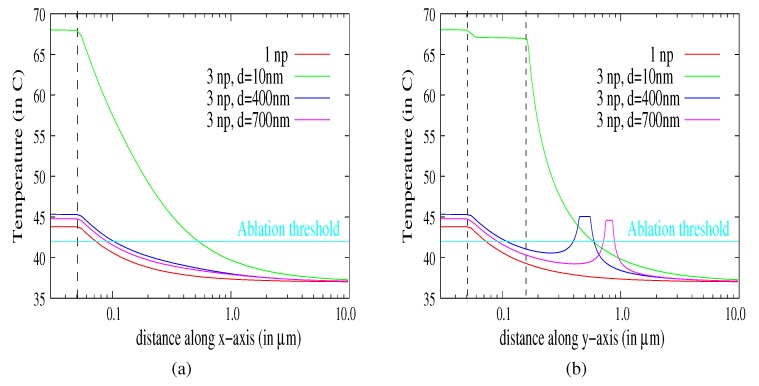
Temperature as a function of the distance to the center of the nanoparticle of diameter $D_{np,1}=100$ nm, for N=1 and a combination of N=3 identical nanoparticles. The inter-distances are respectively $d_{1,2}=d_{1,3}=10$ nm, $d_{1,2}=d_{1,3}=400$ nm, $d_{1,2}=d_{1,3}=700$ nm. The temperature is plotted along the x-axis (**a**) and along the y-axis (**b**).

For a small distance between the nanoparticles (i.e., $d_{1,2}=d_{1,3}=10$ nm, Figure 7a), the maximum temperature increases and is greater than *N* times the temperature elevation induced by three uncoupled particles. $\Delta T_{3np,max}=\left(T_{3np,max}-T_{0}\right)\geq N\Delta T_{np,max}$ (with $\Delta T_{np,max}=7.5{\;}^{\circ }$C; see Figure 2). As expected, the temperature elevation is increased for a short distance between the nanoparticles (i.e., both active electric and thermic coupling between nanoparticles). For a distance larger than $d_{elec}$ (here $d_{1,2}=d_{1,3}=400$ nm, Figure 7b), the maximum temperature elevation decreases, but remains greater than the temperature elevation induced by a single particle $\Delta T_{3np,max}\geq \Delta T_{np,max}$. Increasing $d_{i,j}>d_{therm}$ leads to convergence to $\Delta T_{np,max}$. The nanoparticles are decoupled, and each of them behaves like a single particle. The temperature in the cell is shown in Figure 8a,b.

The coupling between dispersed nanoparticles is weak for an inter-distance greater than 400 nm. In the considered cases (400, 700) nm, the temperature is close to that for a single nanoparticle. We can note that Figure 8b shows the temperature evolutions along the y-axis (configuration of Figure 7b) where the positions of the nanoparticles are $p_{1}\in [-50$; 50] nm and $p_{2}\in [450$; 550] nm for cluster 400 nm and $p_{1}\in [-50$; 50] nm and $p_{2}\in [750$; 850] nm for cluster 700 nm. The temperature profiles are increasing conformally to the presence of the nanoparticles acting like local heat sources.

Hence, the electromagnetic and thermic coupling in the case of the aggregate has a tremendous influence on the temperature elevation: the TACL is larger than eight-times that for a single particle. This could explain the efficiency of the thermal ablation for lower laser power. It has to be noticed that the dispersion of particles in the cell would get them closer to the edges of the cell. The critical distance has been evaluated as 50–100 nm for a single particle (Section 3.2) and can reach 400–800 nm for aggregates.

### 3.5. Discussion of Material Parameters and Configuration

For application in cancer therapy, the illumination wavelengths must be in the biological window (800–1000 nm). By increasing the incident wavelength, the imaginary part of the permittivity of the gold nanoparticle is slightly increasing and requires designing the particle shape or decreasing the laser power [28]. We can note that our results have been compared to the experimental results of [6,7,8,9,10]. Our results are in agreement with the experimental ones (the absorption efficiencies due to the shape of the nanoparticles are counterbalanced by smaller laser power densities producing compatible heat sources *Q* and temperature elevations). Moreover, the treatment of larger cells (typically, a cell size is varying from 1–100 μm [29]) or a mix of a smaller/larger volume of nanoparticles, smaller/greater power can open the way to a process inducing both a controlled local elevation of the temperature above the ablation threshold and ensuring no temperature elevation in the neighboring cells. The increase of the number of nanoparticles, if aggregated, has the similar effect of the increase of the size of the nanoparticle. Indeed, each nanoparticle plays the role of a local temperature source. Therefore, multiple local thermic sources can be induced at the same time, and a global thermic field can also be increased due to the aggregated nanoparticles.

## 4. Conclusions

In this paper, we focus on the analysis of the thermal ablation characteristic length of nanoparticles embedded in cells. We show that the tuning of the temperature elevation and of the distance of action of a single nanoparticle is weakly dependent on the particle size and on the laser power. We propose a method of analysis of numerical data that is based on the medical constraints for thermal ablation. Aggregates of nanoparticles are much more efficient, but represent a potential danger to the surrounding cells. Fortunately, by tuning the laser power, the thermal ablation characteristic length can be controlled. In this study, only spherical particles and cells are considered, and continuous illumination was used. However, the finite element method with the adaptive remeshing is a general method that can handle more complex and more realistic systems (shapes, sizes, materials), including time-dependent evolution of the temperature from a laser pulse.

## Figures and Tables

**Table 1:** Values of the parameters for the photothermal model for the two media (cell, gold nanoparticle) with $j^{2}=-1$.

	ρ (kg·m${}^{-3}$)	$C_{p}$ (m${}^{2}\cdot$s${}^{-2}\cdot$K${}^{-1}$)	κ (kg·m·s${}^{-3}\cdot$K${}^{-1}$)	$\epsilon_{r}$ (at 830 nm)
cell	1090	2185	1.20	2.04
Au	19,300	129	310	−26.61 + j1.67
